# The landscape of research on ferroptosis under hypoxic conditions: a bibliometric analysis

**DOI:** 10.3389/fphar.2025.1519000

**Published:** 2025-03-26

**Authors:** Di Yu, Yibo Hu, Meijuan Ma, Wenjia Li, Xiaohui Zhao

**Affiliations:** ^1^ Department of Basic Medical Sciences, Qinghai Unversity Medical College, Xining, Qinghai, China; ^2^ Department of Orthopaedic Trauma, The Affiliated Hospital of Qinghai University, Xining, Qinghai, China

**Keywords:** ferroptosis, hypoxia, bibliometric analysis, hotspot, citespace

## Abstract

**Background:**

Ferroptosis is a newly identified type of iron-dependent cell death that characterized by an increase in intracellular iron ions, which disrupt the balance of the cellular lipid peroxidation system, causing lipid peroxidation and ultimately resulting in cell death. Interestingly, ferroptosis is modulated by hypoxia and plays a role in hypoxia-related diseases. Therefore, we performed a bibliometric review of the Web of Science Core Collection (WoSCC) database to investigate the link between ferroptosis and hypoxia from January 2013 to December 2023.

**Method:**

The core collection within the Web of Science bibliographic index was consulted to extract relevant articles and reviews. Data on publications, countries, institutions, authors, journals, citations, and keywords in the included studies were systematically analyzed using Microsoft Excel 2019 and CiteSpace 6.3.R1 software.

**Result:**

A comprehensive analysis and visualization of 472 research papers on ferroptosis under hypoxic conditions published between 2013 and 2023 revealed emerging research hotspots and trends. Initially, a scarcity of studies existed in this field. However, this was succeeded by a significant increase in research interest in subsequent years, culminating in a peak of 204 publications in 2023. Research in this field focused primarily on the Asian region. Notably, research hotspots include diseases related to hypoxia, treatment therapy and pathogenesis. Among the researchers in this field, Supuran emerged as the most prolific author. Wuhan University was the leading institution in terms of research output, and China was the most prolific country in this area of study. Among the top ten journals ranked by the number of publications, nine were classified as Q1, indicating the high level of credibility of these studies. The research conducted by Stockwell et al., featured in the journal “*Cell*,” currently has the most citations. Present scholarly pursuits are primarily focused on comprehending the mechanisms through which interventions affect hypoxia-related diseases through the ferroptosis pathway, as well as on probing and pinpointing prospective treatment targets.

**Conclusion:**

This study highlights key areas of interest and emerging trends in ferroptosis research in the presence of hypoxic conditions, thus providing valuable insights for future directions of exploration for the diagnosis and treatment of hypoxia-related diseases.

## 1 Introduction

Ferroptosis is a recently identified mechanism of cell death contingent on iron, which arises from the buildup of iron-catalyzed lipid peroxidation (Liang et al., 2022). Studies have indicated that the low molecular weight compound erastin triggers ferroptosis-specific cell death in tumor cells harboring the mutated oncogene RAS, this process is distinct from apoptosis, necrosis, and autophagy (Li et al., 2020a). The hallmarks of ferroptosis include the build-up of iron within cells and oxidation of lipids, which is predominantly linked to the accumulation of intracellular iron, exhaustion of glutathione (GSH), as well as the deactivation of GSH peroxidase 4 and escalation of lipid peroxidation (Ursini and Maiorino, 2020). A growing body of research has affirmed the association of ferroptosis with various signaling pathways and its involvement in the modulation of numerous diseases (Jiang et al., 2021; Stockwell et al., 2020).

Reduced oxygen availability in tissues, termed hypoxia, affects several cellular processes in diverse cell types and is linked to both normal physiological states and disease conditions, including altitude-induced effects, solid tumor growth, and organ ischemia (McClelland and Scott, 2019; Chen et al., 2022; Ow et al., 2018). Hypoxia modulates proteins involved in iron metabolism, thereby influencing iron levels and lipid peroxidation rate (Fuhrmann and Brune, 2022). Hypoxia-inducible factor-1, modulated by hypoxic conditions, facilitates increased iron absorption, which in turn affects susceptibility to ferroptosis (Gao et al., 2023). Hypoxia also controls ferroptosis through the Nrf2/HO-1 and p62/Keap1/Nrf2 signaling pathways (Cao et al., 2022; Li et al., 2020b). In addition, epigenetic alterations, including microRNAs (miRNAs), long non-coding RNAs (lncRNAs), and methylation, contribute to the regulation of ferroptosis under hypoxic conditions (Yang et al., 2022; Dong et al., 2022; Liu et al., 2023). Accumulating evidence suggests that ferroptosis plays an important role in hypoxia-related diseases such as ischemia-reperfusion (I-R), ischemiastroke (IS), hypoxic tumors, Coronavirus disease-19 (Gao et al., 2023).

Bibliometrics uses mathematical and statistical techniques to collect data on publication rates, characteristics, and patterns in a specific field (Ahmad et al., 2020). Through the analyses of authors, co-occurrences, and citations, research trends and statuses in specific fields can be elucidated. Such research can help determine the productivity of researchers, internal collaboration, and distribution, as well as those of affiliated institutions and journals. Bibliometric studies can uncover focal points of research within a discipline and indicate potential avenues for future studies (Saeed et al., 2024; Agathokleous and Calabrese, 2024). Therefore, this study comprised a bibliometric review of the literature on ferroptosis under hypoxic conditions published between 2013 and 2023, thoroughly examining the prevailing research landscapes, focal areas, and emerging trends. The objective was to identify journal publications, collaborators, keywords, and research trends that can enhance our understanding of diseases, mechanisms, and treatments. Collectively, these findings provide valuable insights for future studies.

## 2 Material and methods

### 2.1 Data sources and search strategy

**FIGURE 1:**
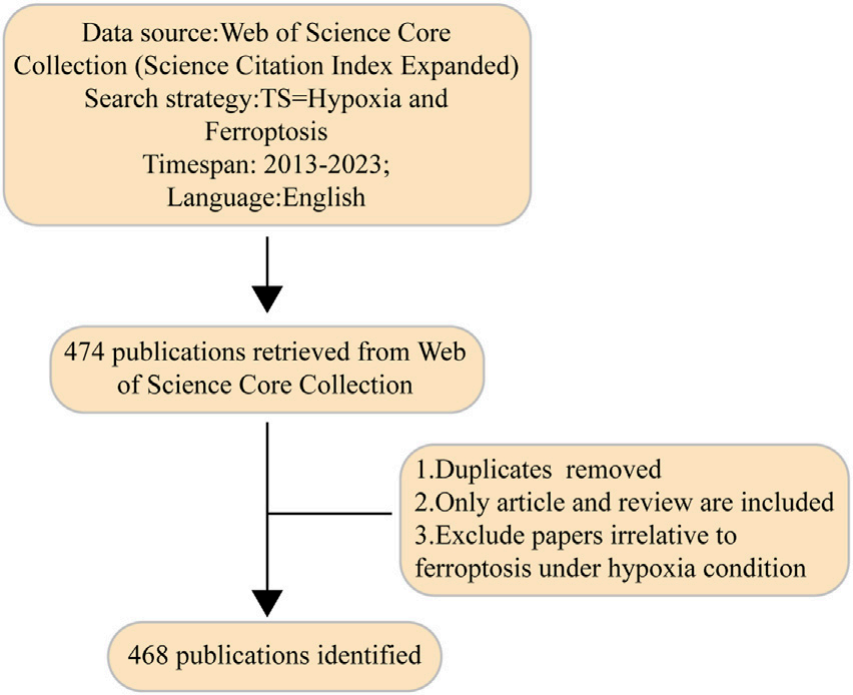
Flow diagram showing the screening process.

Web of Science was selected as the primary database for this study due to its comprehensive coverage of over 12,000 academic journals and its frequent usage by researchers. When compared to other databases such as Scopus, Medline, and PubMed, Web of Science provides the most comprehensive and reliable bibliometric analysis (Zhang et al., 2024; Jiang et al., 2023). So we determined that the Web of Science Core Collection (WoSCC) was the optimal database for bibliometric evaluation in our study. Consequently, we conducted a comprehensive literature search on ferroptosis under hypoxic conditions using the Science Citation Index Expanded function of the WoSCC database, spanning 2003–2023. The process of searching and downloading data for all documents was accomplished in a single day to avoid any bias that may have resulted from the regular updates of the database. The search criteria and methods we used were as follows: Title search equals (Ferroptosis and Hypoxia) AND Language equals (English), with a defined time frame from 1 January 2013 to 31 December 2023. To confirm that the collected publications were pertinent to the primary subject of our research, we scrutinized and documented each publication by examining its title and summary, excluded articles from low-quality or predatory journals. The exclusion criteria were as follows: (1) duplicate publications; (2) entries with missing information such as author names, journals, or publication dates; and literature types categorized as articles and reviews. An expansive flow diagram of the screening process is shown in Figure 1.

### 2.2 Data analysis and visualization

After screening the manuscripts, we extracted data from relevant publications, including titles, keywords, countries or regions, authors, institutions, journals, and the number of citations. Full records and cited references of the retrieved articles were downloaded from the WoSCC database. The data were then converted to TXT format and imported into CiteSpace 6.3.R1 for bibliometric analysis (Chen and Song, 2019).

### 2.3 Bibliometric analysis

We performed bibliometric analysis of several literature characteristics, including the number of publications, countries/regions, institutions, authors, journals, co-cited references, and keywords with the strongest citation bursts. Using CiteSpace, we constructed visual networks of various countries or regions and institutions based on collaborative or co-occurrence data. This tool facilitates the discovery of novel trends and developments in a research field, particularly in the analysis of literature citations and keywords. We used CiteSpace for co-citation analysis of keyword burst detection to identify the current status and hotspots of research on ferroptosis under hypoxic conditions and to predict future research directions in this field.

## 3 Results

### 3.1 Annual publication trends

**FIGURE 2:**
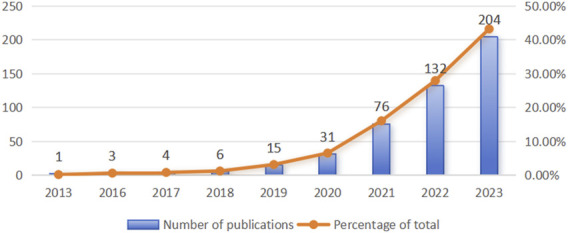
Publication count in the field of ferroptosis under hypoxic conditions from 2013 to 2023.

Between 2013 and 2023, a total of 472 documents on ferroptosis associated with hypoxia were published. The concept of ferroptosis was first introduced by Dixon et al., in 2012, and research on ferroptosis began to emerge in 2013, including studies on ferroptosis under hypoxic conditions. As depicted in Figure 2, the publication count was modest and steady from 2013 to 2018, oscillating annually from one to six papers. Following this period, a notable increase in the number of publications was observed from 2019 to 2023, culminating in 204 articles in 2023. These results provide a promising outlook for the ongoing growth and advancement of this research field.

### 3.2 Analysis of geographical distribution of publications

**FIGURE 3:**
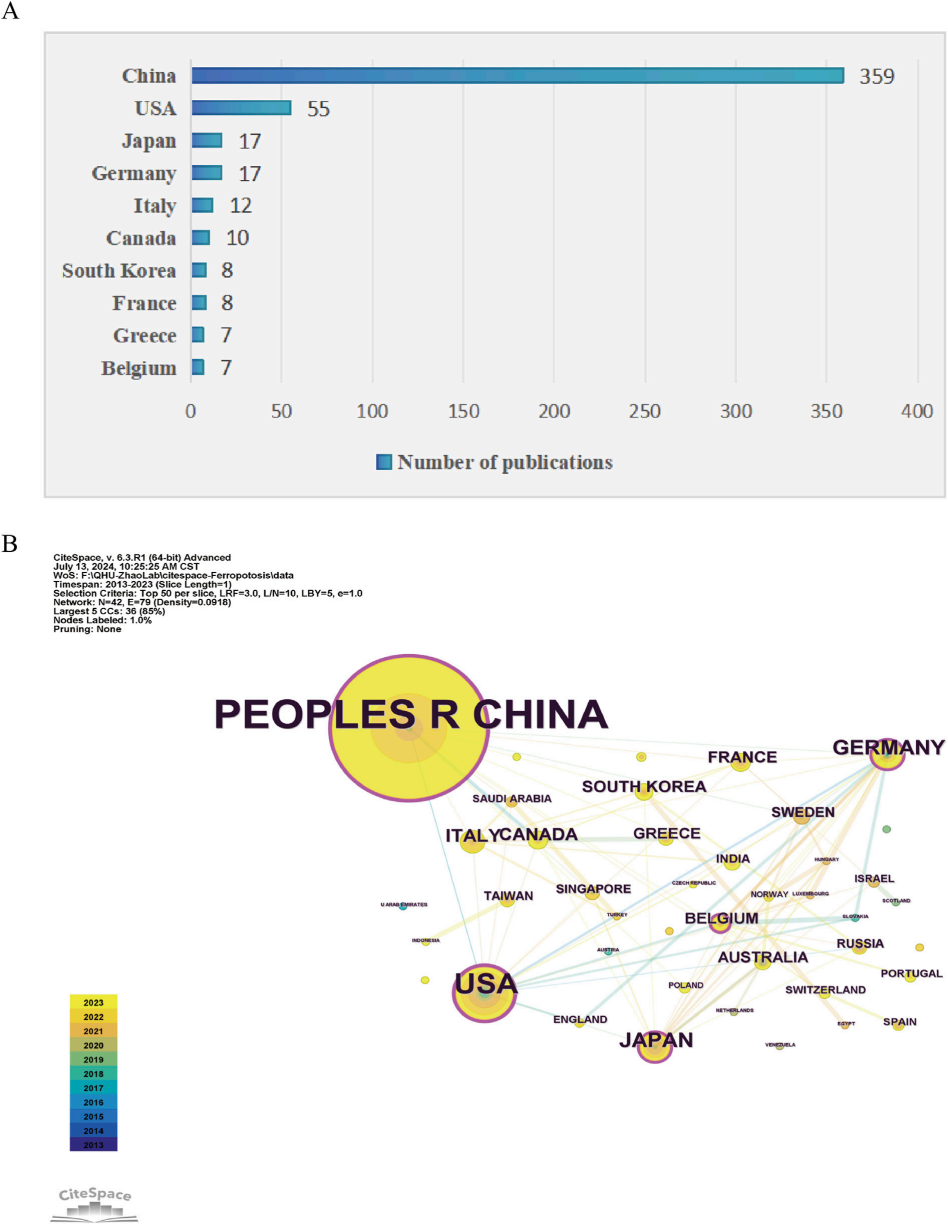
Analysis of geographical distribution of publications. **(A)** Top ten countries/regions with the most published research on ferroptosis under hypoxic conditions. **(B)** Visualization of cooperation among countries/regions where publications related to ferroptosis under hypoxic conditions were visualized using CiteSpace. The lines connecting countries/regions signify collaborative engagements. The largest cluster (green dots) included countries such as China and the United States, and cooperation was primarily focused on these.

As shown in Figure 3A, 42 countries participated in the studies analyzed, with six countries publishing more than ten studies each. China led the tally with the highest number of publications (n = 359), followed by the United States (n = 55), Japan (n = 17), and Germany (n = 17). CiteSpace analysis was also used to visualize country collaborations. As shown in Figure 3B, this network was composed of 42 nodes and 79 links, signifying collaborative ties among China, the United States, Germany, Japan, and Italy. Notably, as show in Table 1, China, the United States, and Germany were identified as the most pivotal participants based on their centrality scores (0.24, 0.21, and 0.17, respectively). The evaluation of both publication volume and centrality indices highlights the pivotal role of China, the United States, Germany, and Japan as leading forces in this research field.

**TABLE 1:** Top 10 prolific countries and institutions in the field of ferroptosis under hypoxic conditions.

Rank	Country	Count	Centrality	Institute (country)	Count	Centrality
1	CHINA	359	0.24	Wuhan University (China)	23	0.04
2	USA	55	0.21	Chinese Academy of Sciences (China)	19	0.47
3	GERMANY	17	0.17	Sun Yat Sen University (China)	17	0.15
4	JAPAN	17	0.2	Shanghai Jiao Tong University (China)	15	0.11
5	ITALY	12	0.07	Central South University (China)	14	0.02
6	C ANADA	10	0.07	Nanjing University (China)	14	0.16
7	FRANCE	8	0.02	Soochow University (China)	13	0.04
8	SOUTH KOREA	8	0.07	Fudan University (China)	13	0.07
9	GREECE	7	0	Southern Medical University (China)	12	0.04
10	AUSTRALIA	7	0.07	Anhui Medical University (China)	11	0.05

### 3.3 Visual analysis of institutions and authors

**FIGURE 4:**
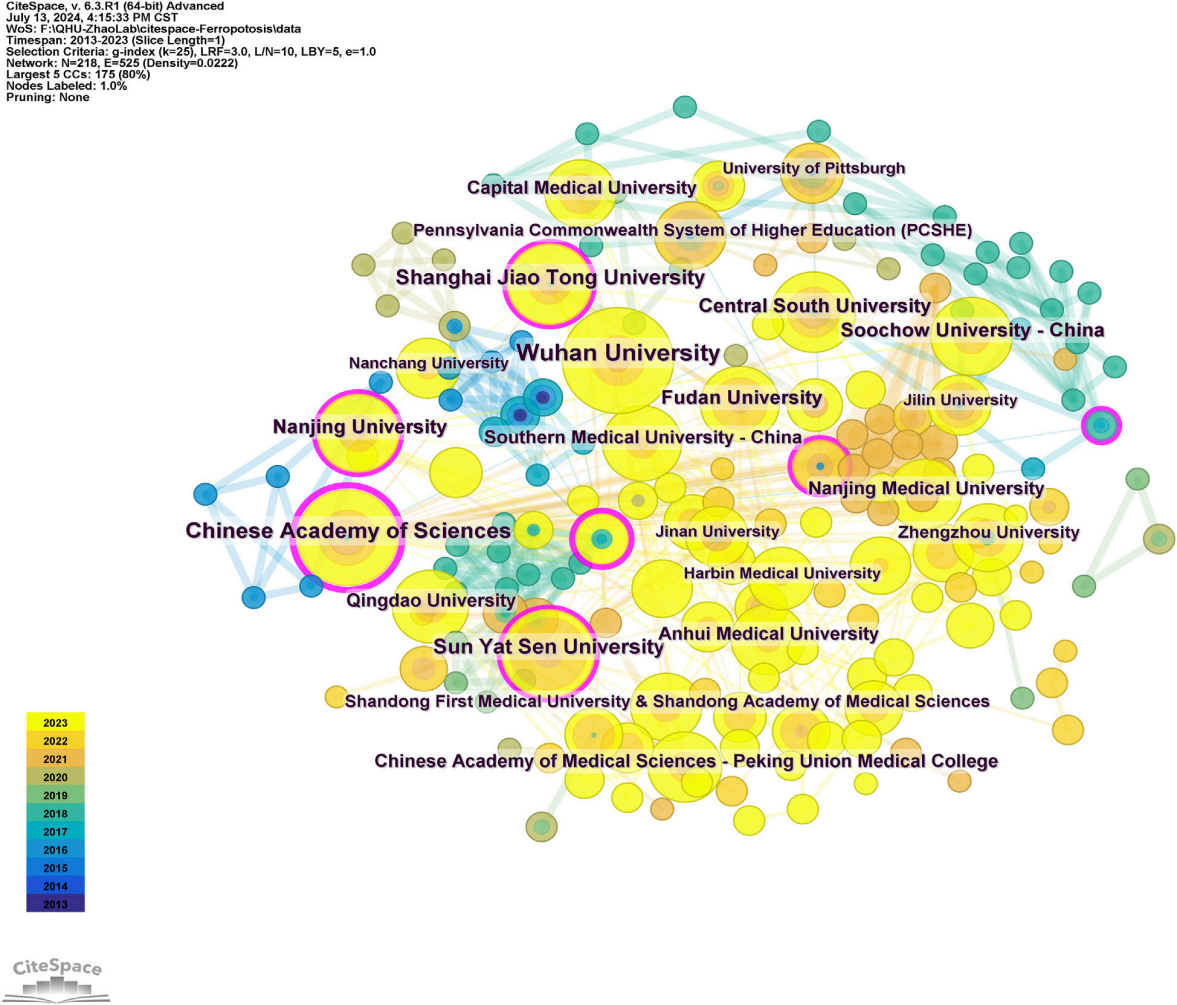
Robust cooperative relationships between institutions.

A total of 218 institutions were involved in the publication of research papers, with 38 of these contributing a minimum of five papers each. The top ten institutions each generated at least 11 papers each (Table 1). Wuhan University published the most articles at 23. The mapping of collaborative institutions, as depicted in Figure 4, revealed a network of 218 nodes interconnected by 525 links, signifying robust cooperative relationships. Wuhan University, Chinese Academy of Sciences, Sun Yat-sen University, Shanghai Jiao Tong University, and Central South University were identified as the most collaborative institutions, demonstrating extensive contributions and strong cooperative bonds among Chinese research institutions in this field. Notably, the Chinese Academy of Sciences had the highest ranking in terms of centrality.

A total of 390 authors were engaged in research involving ferroptosis under hypoxic conditions. The eight most prolific authors along with their related information are presented in Table 2. The top eight authors collectively published 33 articles. Supuran was at the forefront with four articles, followed by Tang, Daolin, Pissas, Georgios, Kang, Rui, Beharier, Ofer, Eleftheriadis, Theodoros, Liakopoulos, Vassilios, Stefanidis, and Ioannis, each having published three articles. These distinguished authors were associated with six distinct research institutions.

**TABLE 2:** Top eight authors with published research on ferroptosis under hypoxic conditions.

Rank	Author	Institute	Count	Centrality
1	Supuran, ClaudiuT	Florence University	4	0
2	Tang, Daolin	Guangzhou Med Univ	3	0
3	Pissas, Georgios	Thessaly University	3	0
4	Kang, Rui	UT Southwestern Med Ctr	3	0
5	Beharier, Ofer	Pittsburgh University	3	0
6	Eleftheriadis, Theodoros	Thessaly University	3	0
7	Liakopoulos, Vassilios	Thessaly University	3	0
8	Stefanidis, Ioannis	Thessaly University	3	0

### 3.4 Visual analysis of journals

The dataset comprised 472 articles from 266 journals. The *International Journal of Molecular Sciences* had the maximum number of publications, totaling ten articles, followed by *Biomedicine and Pharmacotherapy*, *European Journal of Pharmacology*, *Frontiers in Cell and Developmental Biology*, and *Frontiers in Oncology*, each with nine articles. A group of 24 journals published more than five articles, totaling 161 articles. The ten leading journals, ranked by volume of publications, are presented in Table 3. Among the top ten journals, nine were classified as Q1, signifying a high level of academic credibility.

**TABLE 3:** Top 10 journals in the field of ferroptosis under hypoxic conditions.

Rank	Journal	Count	IF (2023)	JCR (2023)
1	Intermational journal of molecular sciences	10	6.208	Q1
2	Biomedicine and pharmacotherapy	9	7.5	Q1
3	European journal of pharmacology	9	5.0	Q1
4	Frontiers in cell and developmental biology	9	5.5	Q1
5	Frontiers in oncology	9	4.7	Q2
6	Acs applied materials and interfaces	8	9.5	Q1
7	Free radical biology and medicine	8	7.4	Q1
8	Cell death and disease	7	9.0	Q1
9	Frontiers in pharmacology	7	5.6	Q1
10	Redox biology	7	11.4	Q1

### 3.5 Analysis of the co-cited representative literature

Table 4 displays the top ten most-cited original articles on ferroptosis in hypoxia research: half for articles and half for reviews. The top ten cited articles were all published in Q1 journals, indicating that their IF and credibility were high. Moreover, the prominence of these studies, all of which were published between 2017 and 2021, highlights the fact that ferroptosis in hypoxia is an emerging research field. The top ten publications collectively received over 418 citations. The most frequently cited article was that of Stockwell et al., which appeared in the journal *Cell*, garnering 73 citations. This review elucidates the mechanisms underlying ferroptosis and their connection to other areas of biology and medicine regarding this emerging form of regulated cell death (Stockwell et al., 2017).

**TABLE 4:** Top 10 most cited original articles on ferroptosis in hypoxia research.

Rank	Cited number	Title	Type	Year	Journal	IF (2023)	JCR (2023)	References
1	73	Ferroptosis: A Regulated Cell Death Nexus Linking Metabolism, Redox Biology, and Disease	Review	2017	Cell	64.5	Q1	Stockwell et al. (2017)
2	64	Ferroptosis as a target for proection against cardiomyopathy	Article	2019	PNAS	11.1	Q1	Fang et al. (2019)
3	61	Ferroptosis: mechanisms, biology and role in disease	Review	2021	Nat Rev Mol Cell Biol	112.7	Q1	Jiang et al. (2021)
4	56	Ferroptosis: past, present and future	Review	2020	Cell Death Dis	9.0	Q1	Li et al. (2020a)
5	43	FSP1 is a glutathione-independent ferroptosis suppressor	Article	2019	Nature	64.8	Q1	Doll et al. (2019)
6	40	The CoQ oxidoreductase FSP1 acts parallel to GPX4 to inhibit ferroptosis	Article	2019	Nature	64.8	Q1	Bersuker et al. (2019)
7	38	Broadening horizons: the role of ferroptosis in cancer	Review	2021	Nat Rev Clin Oncol	78.8	Q1	Chen et al. (2021)
8	37	Ferroptosis: molecular mechanisms and health implications	Review	2020	Cell Res	44.1	Q1	Tang et al. (2020)
9	36	ACSL4 dictates ferroptosis sensitivity by shaping cellular lipid composition	Article	2017	Nat Chem Biol	14.8	Q1	Doll et al. (2017)
10	33	Hypoxia inhibis frritinophagy, increasesmitochondrial fritin, and proectsfrom ferroptosis	Article	2020	Redox Biol	11.4	Q1	Fuhrmann et al. (2020)

### 3.6 Keyword analysis

#### 3.6.1 Keyword co-occurrence analysis

Keywords related to ferroptosis under hypoxic conditions were identified and analyzed using the CiteSpace software. The keywords were analyzed over the publication period of 2013–2023. The top five keywords were cell death (n = 104), hypoxia (n = 96), oxidative stress (n = 91), ferroptosis (n = 65), and expression (n = 62). Large centrality values represent more cooperation of one node with other nodes. Centrality analysis revealed that oxidative stress (n = 0.21), ferroptosis (n = 0.18), cell death (n = 0.17), hypoxia (n = 0.12), and expression (n = 0.11) exhibited high centrality and emerged as the most influential keywords.

#### 3.6.2 Keyword cluster analysis

**FIGURE 5:**
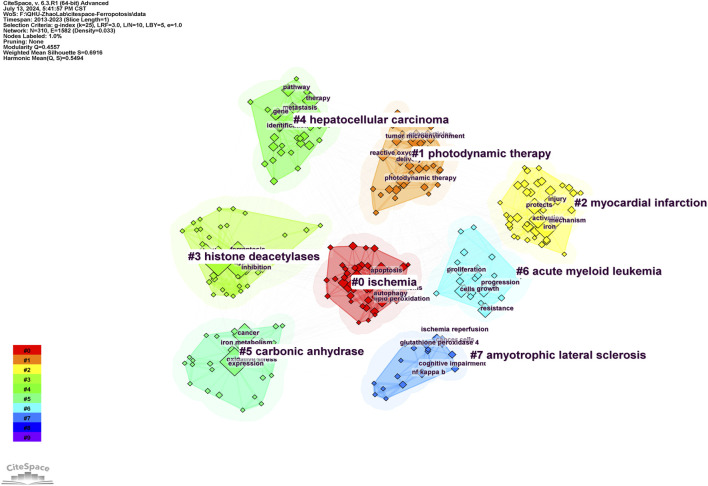
Keyword clustering graph for ferroptosis under hypoxic conditions.

The keyword clustering graph illustrates the structural features of the clusters, highlighting their key nodes and essential connections (Wang et al., 2024). A network map of the keyword clusters was generated using the findings of the co-occurrence analysis. The network map has a rich clustering structure, and the structure is persuasive, as indicated by the modularity Q = 0.4557 > 0.3 and mean silhouette = 0.6916 > 0.5 of the keyword clustering map, both of which denote a well-defined and logical clustering framework that is highly reliable. As depicted in Figure 5, the top eight cluster tag groups (#0–7), which were scrutinized and refined using clustered keywords, encapsulated an overarching research paradigm for studies on ferroptosis under hypoxic conditions. Within these clusters, Cluster 0 (red) displayed topics on ischemia, Cluster 1 (orange) concentrated on photodynamic therapy, Cluster 2 (yellow) was concerned with myocardial infarction, and Cluster 3 (light green) focused on histone deacetylases. Cluster 4 was dedicated to hepatocellular carcinoma research, Cluster 5 (dark green) was centered on carbonic anhydrases, Cluster 6 (light blue) was centered on acute myeloid leukemia, and Cluster 7 (blue) was dedicated to amyotrophic lateral sclerosis.

#### 3.6.3 Keyword citation burst analysis

**FIGURE 6:**
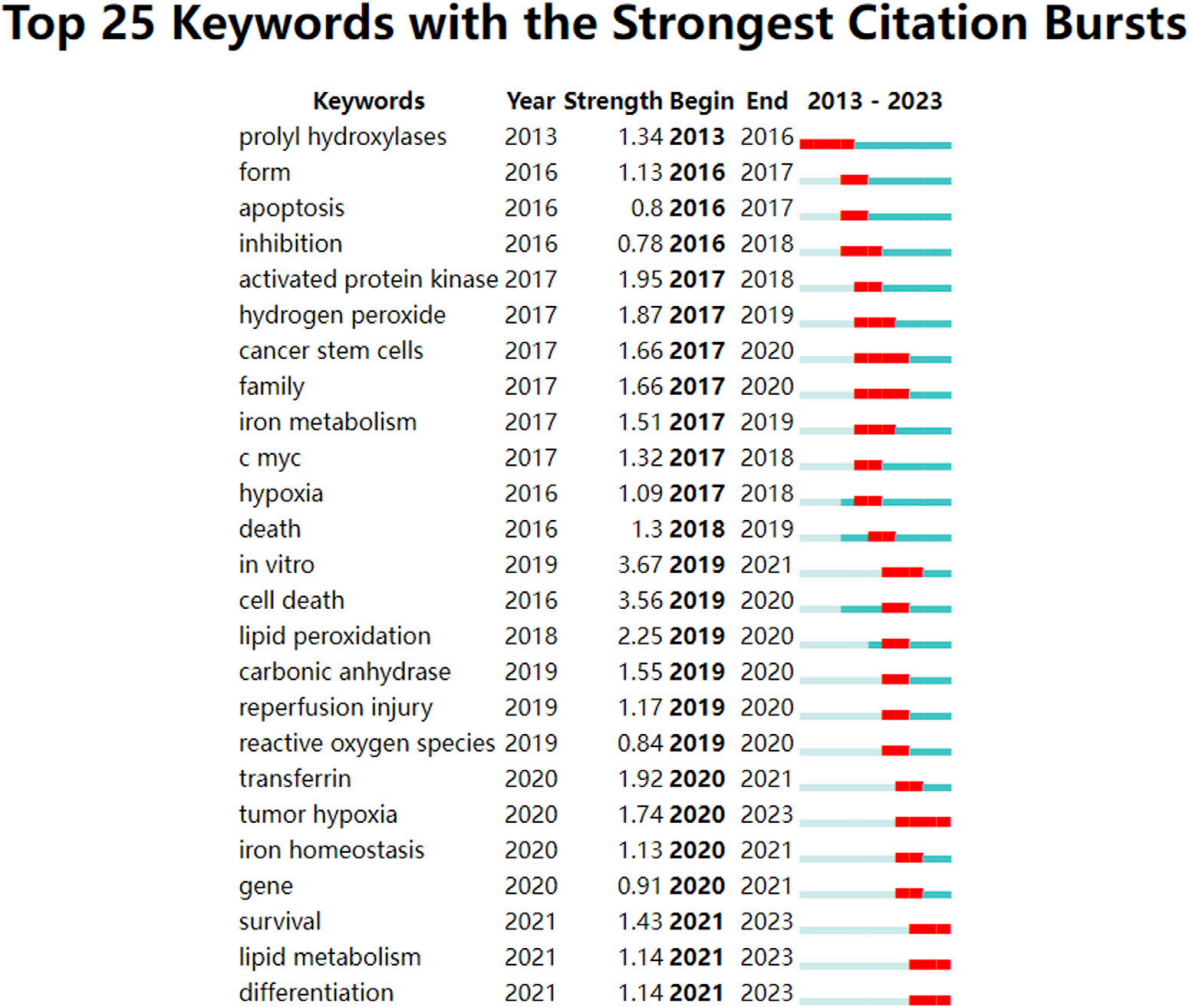
Keywords with intense citation bursts in the field of ferroptosis under hypoxic conditions.

Analysis of keyword citation bursts can reveal research trends over a defined period (Zhang et al., 2024). Using an analysis of keyword co-occurrence, a burst analysis of keyword citations was conducted to identify the top 25 most-cited terms, as shown in Figure 6. In this burst analysis, “Begin” and “End” indicate the time of the burst. “Strength” refers to the strength of a burst and represents credibility over time. By concentrating on terms with pronounced surge rates, trending subjects in this research field can be identified by researchers.

The keyword citation burst analysis delineates that encompassing the years 2013–2017 spotlights terms such as prolyl hydroxylases, apoptosis, inhibition, activated protein kinase, iron metabolism, and c-myc, which predominantly address alterations in physiological processes and underlying molecular dynamics. From 2018 to 2020, terms such as lipid peroxidation, reperfusion injury, reactive oxygen species, transferrin, tumor hypoxia, iron homeostasis, and genes are focused in, thereby highlighting the repercussions of ferroptosis under hypoxic conditions. From 2021 to 2023, the terms survival, lipid metabolism, and differentiation are commonly observed, thus emphasizing the influence and underlying mechanisms of various diseases. The keywords from 2021 to 2023 remain relevant today and represent emerging trends and future research directions.

## 4 Discussion

### 4.1 General overview

Over the past 10 years, a significant increase in academic attention and investigative work on ferroptosis under hypoxic conditions can be noted, culminating in an expanding volume of studies annually. This quantitative literature review used CiteSpace to analyze and portray 472 papers on this subject to uncover critical research hotspots and trends. In 2012, a novel molecular entity, erastin, was shown to enhance peroxide accumulation during Fe2+ synthesis, leading to intracellular mitochondrial atrophy and increased membrane density. This unique mechanism results in a distinct form of cell death, known as ferroptosis (Dixon et al., 2012). Initial research on ferroptosis was limited. However, from 2018 to 2020, a marked increase in the volume of publications was recorded, signifying an expanded scope and depth of research. After 2020, a swift increase in this trend was noted. This pattern highlights the vast potential of ferroptosis under hypoxic conditions, which is garnering increasing interest from researchers. Therefore, this field of study is expected to continue to increase in popularity in coming years.

Within the ranking of the top ten countries contributing to the literature in this research field, nine were developed countries, with China being the sole developing country. However, China had the highest number of publications and its centrality score (0.24) was the highest, indicating benign connections with developed countries in the field.

An analysis of the institutional cooperation network revealed that institutions in China, such as Wuhan University, Chinese Academy of Sciences, Sun Yat Sen University, Shanghai Jiao Tong University, and Central South University, were leading in research on ferroptosis under hypoxic conditions. The top eight scholars listed in Table 2 were largely associated with developed countries. The research group of Supuran at the University of Florence focused primarily on the conception of groundbreaking treatment plans for malignant solid tumors, aiming to counteract the progression and drug resistance induced by hypoxic and acidic conditions within tumors. Their investigative work has revealed that the use of carbonic anhydrase inhibitors potentiates ferroptosis, leading to a significant reduction in tumor expansion (Nerella et al., 2022; Yusuf et al., 2022; Supuran, 2021). Tang et al. elucidated a pioneering molecular process underlying sorafenib resistance and proposed that MT-1G functions as an emergent modulator of ferroptosis in hepatocellular carcinoma cells (Sun et al., 2016). Furthermore, they systematically summarized the principal molecular underpinnings of ferroptosis; outlined the interactions between ferroptosis and cancer-related signaling pathways; and explored the possible therapeutic uses of ferroptosis in systemic therapy, radiotherapy, and immunotherapy (Chen et al., 2021; Tang et al., 2021).

Eleftheriadis was primarily committed to investigating the underlying processes of ischemia-reperfusion (I-R) damage and is pursuing efficacious treatment methods aimed at forestalling or mitigating I-R lesions. His research has shown that during I-R injury, periods of oxygen deprivation and subsequent reoxygenation lead to upregulation of the enzyme IDO, also known as indoleamine 2,3-dioxygenase 1. This upregulation triggers apoptosis through GCN2K and ferroptosis through the activation of aryl hydrocarbon receptor (Eleftheriadis et al., 2021a). Furthermore, he discovered that the aryl hydrocarbon receptor becomes active during the reoxygenation phase, causing the generation of reactive oxygen species, lipid peroxidation, and cell death through ferroptosis (Eleftheriadis et al., 2021b). Meanwhile, Myocardial Ischemia-Reperfusion Injury (MIRI) occupies an important position in ischemia-reperfusion (I-R) injury. Many studies have also discovered the relationship between ferroptosis and MIRI. MIRI is a type of cardiac dysfunction commonly seen in conditions like acute myocardial infarction and coronary heart disease (Xiang et al., 2024). During this process, hypoxia due to ischemia leads to calcium overload, which in turn causes mitochondrial dysfunction and subsequently triggers cardiomyocyte ferroptosis (Jiang et al., 2016). Elevated ROS levels promote ferroptosis by triggering the Fenton reaction, decreasing GPX4 and Nrf2 levels, and reducing HIF-1 due to hypoxia. Additionally, hypoxia impairs the mitochondrial electron transport chain (ETC.), leading to higher ROS production and inducing ferroptosis (Zhao et al., 2023). Moreover, over-activation of HIF due to hypoxia upregulates TfR (transferrin receptor) expression, causing iron overload during MIRI and ultimately leading to ferroptosis (Zhang et al., 2019). In summary, calcium overload, excessive ROS, and iron overload driven by HIF all contribute to the occurrence of ferroptosis. Therefore, ferroptosis is intrinsically linked to cardiomyocyte damage in MIRI under hypoxic conditions.

The analysis of publication output and citations revealed that among the top ten journals with the most published articles. The *International Journal of Molecular Sciences* published the most articles in this field; however, it was cited less frequently. *Free Radical Biology and Medicine* and *Redox Biology* published fewer articles, but the relevant articles in these journals were highly cited. *Free Radical Biology and Medicine* published several highly cited articles related to hypoxia and ferroptosis in different diseases, including osteoporosis, acute kidney injury, and myocardial ischemia/reperfusion injury. The journal *Redox Biology* published diverse studies on the association between hypoxia and ferroptosis. An article in this journal reported that under conditions of oxygen deficiency, the process of ferritinophagy is suppressed, whereas the levels of mitochondrial ferritin (FTMT) are augmented and the onset of ferroptosis is averted. These findings facilitate our understanding of the regulatory mechanisms of FTMT under hypoxic conditions and indicate a connection between ferritinophagy and the susceptibility of macrophages to ferroptosis (Fuhrmann et al., 2020). Another study revealed that hypoxia-triggered HIF-1α significantly upregulates lncRNA-PMAN, thereby modulating ferroptosis through the stabilization of SLC7A11 mRNA under hypoxic conditions within GC cells and promotes the formation of peritoneal disseminated tumors *in vitro* (Lin et al., 2022). Furthermore, other prestigious journals such as *ACS Applied Materials and Interfaces* and *Cell Death and Disease* also published valuable contributions to the field of ferroptosis under hypoxia research.

### 4.2 Research hotspots, frontiers, and prospects

The literature citation analysis can effectively reveal research trends, assess academic impact, and construct knowledge structures and research networks. To ensure the accuracy and reliability of the analysis results, we have established a minimum citation threshold to maintain the focus on influential research. Articles with fewer than 25 citations were excluded from our analysis. Additionally, self-citations and citations from the same author are excluded. Our analysis revealed that the review “Ferroptosis: A Regulated Cell Death Nexus Linking Metabolism, Redox Biology, and Disease” by Stockwell et al. published in *Cell* in 2017 received the highest number of citations. This review highlights the mechanisms underlying ferroptosis, its relevance to various fields of biology and medicine, and proposes methodologies and criteria for examining this novel mode of programmed cell death (Stockwell et al., 2017). The article ranking second in citation frequency, titled “Ferroptosis: A Protective Target Against Cardiomyopathy,” featured in the Proceedings of the National Academy of Sciences of the United States in 2019, disclosed that mitochondrial oxidative harm is a predominant factor in heart injury stemming from ferroptosis. Moreover, the use of ferrostatin-1 and iron chelators improved cardiac dysfunction triggered by both sudden and prolonged I-R episodes in murine models, highlighting the potential of ferroptosis-targeted therapies to safeguard against cardiomyopathy (Fang et al., 2019). The article ranking thrid in citation frequency, titled “Ferroptosis: mechanisms, biology and role in disease,” featured in Nat Rev Mol Cell Biol in 2021, it revealed the potential physiological functions of ferroptosis in tumor suppression and immune surveillance, and its pathological roles, including roles of ferroptosis in neurotoxicity and brain injuries. Refers to brain injuries, Ischemic Stroke (IS) occupies an extremely important position in brain injuries due to its high incidence and disability rate (Zhu et al., 2024). IS primarily results from focal cerebral ischemia and hypoxia due to the obstruction of cerebral blood flow (Xu et al., 2023). A study revealed that hypoxia caused by IS upregulates the expression of ferritin and TfR1, which enhances iron uptake by neurons, leading to increased intracellular iron levels and subsequently triggering ferroptosis (Lan et al., 2020). Additionally, the deficiency of ceruloplasmin under hypoxic conditions can disrupt iron metabolism and exacerbate oxidative damage, facilitating iron accumulation-induced ferroptosis (Ryan et al., 2018). It has been reported that elevated glutamate levels under hypoxia can induce ferroptosis by disrupting intracellular iron homeostasis and causing brain injury (Li et al., 2017). Moreover, Oxidative stress induced by hypoxia results in excessive ROS (reactive oxygen species) accumulation, which activates Nrf2 (nuclear factor erythroid 2-related factor 2), thereby increasing GSH, SLC7A11, and GPX4 (glutathione peroxidase 4) to protect cells from ferroptosis in IS(Kwak et al., 2002; Dodson et al., 2019). Meanwhile, the accumulation of glutamate inhibits cystine uptake via the suppression of system Xc−, leading to decreased glutathione (GSH) levels and promoting ferroptosis through the activation of ATF4-mediated ferroptotic genes in IS and increased glutamate under hypoxia also contributes to ROS production, which activates Nrf2 and inhibits ferroptosis (Speer et al., 2013; Shibata and Kobayashi, 2008). The highly cited articles that we have analyzed here had many promoting effects on subsequent research. These articles provide a comprehensive foundational framework, elucidate mechanistic pathways, identify regulatory factors and therapeutic targets, link ferroptosis to pathological conditions, standardize research methods, and highlight future research directions. They have facilitated a deeper understanding of this regulated form of cell death and its impact on human health and disease.

Keywords play a crucial role in identifying emerging trends and guiding future research. The keyword clustering graph shows that ischemia, photodynamic therapy, myocardial infarction, histone deacetylases, hepatocellular carcinoma, carbonic anhydrase, acute myeloid leukemia, and amyotrophic lateral sclerosis were popular topics in this field. Keywords with the strongest citation burst analysis visually represent changes in research focus within this field over a specified period and show both continuity and variability in research hotspots. The top ten emerging keywords in this field were “*in vitro*,” “cell death,” “lipid peroxidation,” “activated protein kinase,” “transferrin,” “hydrogen peroxide,” “tumor hypoxia,” “cancer stem cells,” “family,” and “carbonic anhydrase.” Notably, “*in vitro*” and “cell death” ranked first and second in terms of emergence strength (strength = 3.67 and 3.56, respectively). The keywords“*in vitro*”well reflects the important role of *in vitro* experiments in this field. Significant progress has been made in the study of ferroptosis under hypoxic conditions through *in vitro* experiments, revealing the complex relationship between hypoxia and ferroptosis and providing new insights for the treatment of related diseases. These studies include the regulatory mechanisms of hypoxia on ferroptosis, the inhibition of ferroptosis *in vitro* experiments, the cell type-specific differences in hypoxia-induced ferroptosis and the interplay between ferroptosis and hypoxia in the tumor microenvironment (Zheng et al., 2023; Li et al., 2022; Liu et al., 2024; Li et al., 2024). Future research will further elucidate the molecular mechanisms of ferroptosis under hypoxic conditions, particularly the interactions between the HIF signaling pathway and iron metabolism or lipid peroxidation. Utilizing *in vitro* cell models to study hypoxia-induced ferroptosis will facilitate the development of novel therapeutic strategies targeting ferroptosis, especially in the treatment of neurodegenerative diseases and cancer (Costa et al., 2023; Wang et al., 2021). Moreover, combining gene editing, metabolomics and cell biology techniques will deepen the understanding of the regulatory network of ferroptosis under hypoxic conditions, providing theoretical support for clinical applications. The keyword “cell death” reflects the complex interplay between ferroptosis and hypoxia in cell death mechanisms, emphasizing the coordinated regulation of multiple cell death pathways under hypoxic conditions. Under hypoxic conditions, cell death involves the interplay of multiple cell death pathways. Hypoxia-induced cell death is not only associated with ferroptosis but also closely related to other cell death pathways, such as apoptosis, necroptosis, and pyroptosis, these pathways interact in complex ways (Yang et al., 2021; Lenihan and Taylor, 2013). Notably, there is a significant interaction between ferroptosis and PANoptosis (a composite form of cell death that includes pyroptosis, apoptosis, and necroptosis). Studies have shown that hypoxia-induced cell death can be alleviated by the combined use of ferroptosis inhibitors and PANoptosis inhibitors, indicating that both pathways may be simultaneously activated under hypoxic conditions (Wu et al., 2025). Refers to the key word “lipid peroxidation”, It has been reported that hypoxia caused by COVID-19 can increase circulating ferritin levels, leading to iron deficiency, oxidative stress, and lipid peroxidation, which ultimately result in ferroptosis (Qiu et al., 2024). The keywords “tumor hypoxia,” “cancer stem cells” revealed the high correlation between ferroptosis and cancer. Increasing evidence indicates that cancer development is associated with ferroptosis, which is often suppressed in various cancers, including hepatocellular carcinoma, pancreatic cancer, and gastric cancer. Moreover, the connection between cancer and ferroptosis is closely linked to hypoxia. Many solid tumors exhibit hypoxic regions due to inadequate blood flow (Li et al., 2021). Studies have shown that hypoxia is related to the interplay between SLC7A11 and ferroptosis (Zhang et al., 2018; Badgley et al., 2020). Specifically, hypoxia upregulates HIF-1, which in turn increases ELAVL1 levels. ELAVL1 then binds to SLC7A11, enhancing its expression and thereby promoting cancer cell growth by inhibiting ferroptosis (Lin et al., 2022). Under hypoxic conditions, SLC7A11 is upregulated through the inhibition of METTL14, which reduces reactive oxygen species (ROS) and thereby suppresses ferroptosis, thus facilitating the progression of hepatocellular carcinoma (HCC) (Fan et al., 2021). Additionally, hypoxia enhances the activity of HIF-1 transcription, leading to increased TfR1 and DMT1 expression, which helps cervical cancer (CC) cells resist ferroptosis (Christova and Templeton, 2007). However, the mechanisms underlying the relationship between ferroptosis and cancer under hypoxia are still not fully understood and require further investigation.

Overall, this study used the CiteSpace 6.2.R7 software to visualize and analyze the literature on ferroptosis under hypoxic conditions within the WoSCC database. However, this study had several limitations. First, the analysis only included articles from WoSCC and ignored articles from other databases, such as CNKI and Scopus. In addition, because citations are expected to peak gradually 3–10 years after publication, the current analysis may not have highlighted recently published articles.

## 5 Conclusion

Research on ferroptosis under hypoxic conditions is currently in the early stages of development. Based on current global trends, a significant increase in publications and the number of researchers actively involved in this topic is expected. Notable advancements have been achieved in research on ferroptosis related to hypoxia-related diseases. Significant progress has been made in the study of ferroptosis in relation to hypoxia-related diseases. The focal point of this study was the Asian continent, with China emerging as the most prolific contributor to this area of study. The primary areas of interest were concentrated on deciphering the modulatory mechanisms of hypoxia-associated diseases through the ferroptosis pathway and identifying potential therapeutic targets.
